# Epitope-based precision immunotherapy of Type 1 diabetes

**DOI:** 10.1080/21645515.2022.2154098

**Published:** 2023-01-19

**Authors:** Rebuma Firdessa Fite, Camillo Bechi Genzano, Roberto Mallone, Remi J. Creusot

**Affiliations:** a Columbia Center for Translational Immunology, Department of Medicine, Columbia University Irving Medical Center, New York, NY, USA; b Université Paris Cité, Institut Cochin, CNRS, INSERM, Paris, France; c Assistance Publique Hôpitaux de Paris, Service de Diabétologie et Immunologie Clinique, Cochin Hospital, Hôpitaux Universitaires de Paris Centre-Université de Paris, Paris, France

**Keywords:** Type 1 diabetes, antigen-specific therapy, antigen-specific tolerance, epitope, precision medicine, endotype

## Abstract

Antigen-specific immunotherapies (ASITs) address important clinical needs in treating autoimmune diseases. However, Type 1 diabetes is a heterogeneous disease wherein patient characteristics influence responsiveness to ASITs. Targeting not only disease-relevant T cell populations, but also specific groups of patients using precision medicine is a new goal toward achieving effective treatment. HLA-restricted peptides provide advantages over protein as antigens, however, methods for profiling antigen-specific T cells need to improve in sensitivity, depth, and throughput to facilitate epitope selection. Delivery approaches are highly diverse, illustrating the many ways relevant antigen-presenting cell populations and anatomical locations can be targeted for tolerance induction. The role of persistence of antigen presentation in promoting durable antigen-specific tolerance requires further investigation. Based on the outcome of ASIT trials, the field is moving toward using patient-specific variations to improve efficacy, but challenges still lie on the path to delivering more effective and safer treatment to the T1D patient population.

## GRAPHICAL ABSTRACT

**Figure:**
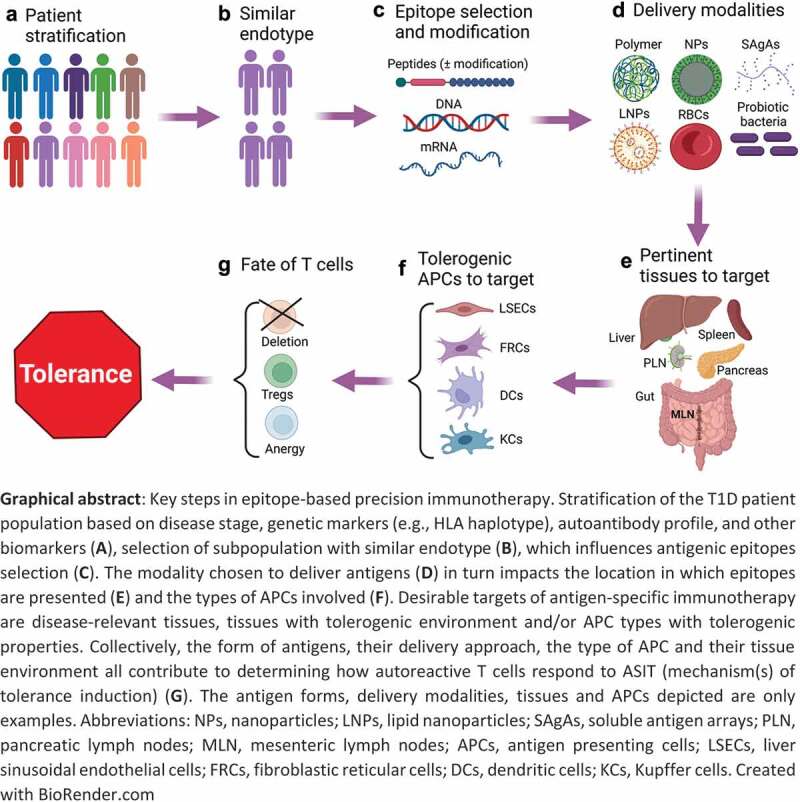

## Type 1 diabetes and the need for antigen-specific therapies

Type 1 diabetes (T1D) is characterized by a progressive loss of insulin-producing β-cells, which is primarily caused by autoreactive CD4+ and CD8+ T cells, as a result of impaired immune tolerance to β-cell antigens. Several genetic factors contribute to the disease in at least two different ways. First, they may increase β-cell fragility (increased oxidative and endoplasmic reticulum stress, sensitivity to proinflammatory cytokines).^1^ Second, they may impair general tolerance mechanisms (as suggested by the occurrence of other autoimmune conditions in both T1D patients^2^ and its animal model, the non-obese diabetic (NOD) mouse.^3^ Administration of exogenous insulin for the last 100 years has saved countless lives; however, it can rarely achieve tight regulation of blood glucose levels, such that episodes of hyper- and hypoglycemia remain commonplace, and their recurrence leads to long-term complications.^4^ Even when perfect glycemic control is achieved, there is still a residual 2- to 3-fold hazard ratio of death from any cause, particularly from cardiovascular disease, in T1D vs. non-diabetic individuals.^5^ Transplantation of pancreatic islets is limited by the paucity of donors and vigorous allogeneic immune responses to grafted islets requiring strong immunosuppression that substantially raises the risk of opportunistic infections, malignancies, and beta-cell toxicity.^6–8^ While the use of autologous stem cell-derived β-cells appears feasible in the future, these cells will remain vulnerable to autoreactive T cells.

**Figure 1.**
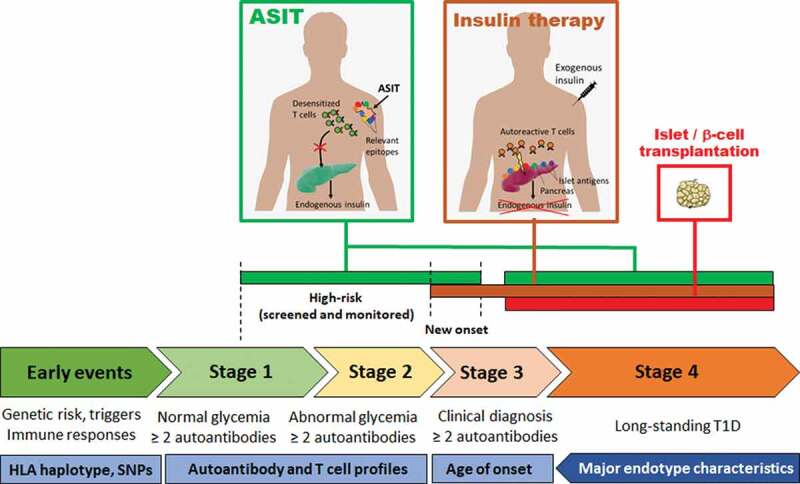
Stages of human T1D disease, corresponding endotype characteristics and windows of opportunities for ASIT.

Targeted immunotherapies aim to disable only relevant elements of the immune system in order to obviate global immunosuppression. Targeting specific immune cells or pathways is mostly about safety and leaving most of the immune system untouched, though this may increase efficacy in some cases. Biologics have supplanted immunosuppressive drugs in the last 10–20 years of clinical trials of T1D treatments, with the removal of specific immune cell types or blockade of specific immune pathways.^9^ While more selective, these approaches still do not make any distinction between autoimmune cells and other immune cells. In contrast, antigen-specific immunotherapies (ASITs) offer a way to selectively engage autoreactive cells and neutralize them through various mechanisms of tolerance. For this to work, however, autoreactive T cells need to be engaged under appropriate conditions that favor tolerance. It is challenging to achieve these conditions in patients, not only because of the underlying local inflammation associated with the disease, but also because of genetic risk factors, some of which impair the induction and maintenance of tolerance (partly explaining how T1D developed in the first place). There are multiple stages of T1D disease progression (Figure 1), and ASITs are useful at stages where there are enough residual β-cells left (or transplanted β-cells) to protect by blocking autoimmune responses. Otherwise, controlled exogenous insulin delivery is required.

**Figure 2.**
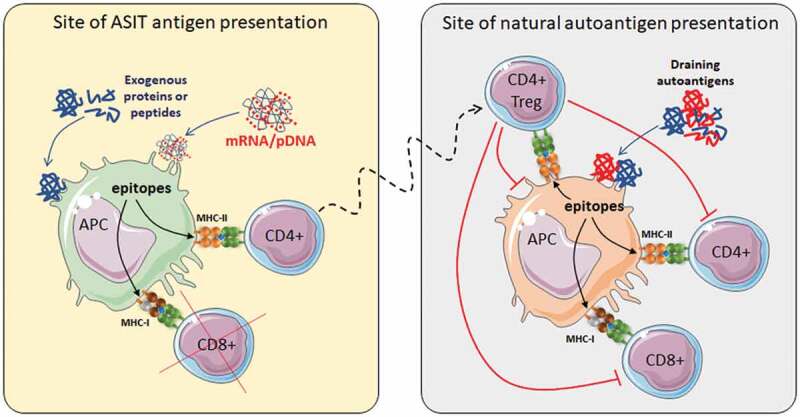
Bystander suppression.

ASITs that induce durable tolerance can be used in high-risk patients, patients with recent onset who have sufficient residual C-peptide (reflecting endogenous insulin production) and, in the future, patients with grafted autologous stem cell-derived β-cells. Patients with allogeneic islet grafts will require additional treatments to curb alloreactivity, although autoreactive Tregs may provide some protection through bystander suppression (Figure 2).

As ASIT cannot achieve full antigen coverage, they rely on bystander suppression as a key mechanism, through which immuno-regulatory T cell populations (mostly CD4+ Tregs) induced under appropriate conditions (left) can migrate to disease-relevant sites (e.g., pancreatic lymph nodes and pancreas; right), find immunogenic APCs that present their epitopes, downregulate the stimulatory function of these APCs and suppress autoreactive T cells that are concomitantly engaged by these APCs and/or prevent later stimulation by these APCs. As the diversity of epitopes presented by these APCs increases, the diversity of autoreactive T cells suppressed also increases. If APCs at these sites all present a very diverse set of epitopes, fewer epitopes may be needed for ASIT. Infectious tolerance is achieved when suppressed T cells themselves are turned into regulatory T cells, which is a difficult yet ideal scenario to achieve.

## Why a need for precision therapy as well?

Precision therapy is a therapeutic approach that is tailored to an individual or group of patients according to the specific biological and molecular features of the disease (e.g. targeting a specific gene mutation in cancer). It constitutes another aspect of targeted therapy that is more about efficacy and maximizing responsiveness by taking the unique characteristics of patients into account.^10^ Some patients go through the different stages of disease progression (Figure 1) quite rapidly and are often diagnosed at a younger age, while other patients progress more slowly and develop diabetes well into adulthood. As T1D is both a multifactorial and polygenic autoimmune disease, it is likely that the number of genetic risk factors and the exposure to environmental influences and triggers, in combination, dictate the pace of disease progression. Due to this complexity, the factors that drive rapid versus slow progression remain poorly understood. Patients are also characterized by different autoantibody (AAb) profiles, with four major AAbs (against insulin, GAD65, IA-2 and ZnT8) being detectable in the serum prior to the onset of diabetes.^11^ Individuals with two or more AAbs have a high risk of progressing to clinical T1D and those with one are at an intermediate risk.^11^ Among genetic risk factors, the HLA class II locus shows the greatest association with T1D. For example, HLA-DRB1*04:01/2/4/5 (HLA-DR4, often inherited in linkage disequilibrium with HLA-DQB1*03:02 (HLA-DQ8)) and HLA-DRB1*03 (HLA-DR3, often linked with HLA-DQB1*02 (HLA-DQ2)) are most frequently found in T1D patients of European descent.^12,13^ HLA haplotypes, both class I and class II, limit the number of peptides that can be presented for each antigen, and it is highly probable that key epitopes are preferentially presented by those aforementioned HLA-DR and DQ haplotypes. Moreover, early onset was associated with an AAb profile dominated by anti-insulin (primarily in individuals with at least one allele of HLA-DR4/DQ8 haplotype), whereas later onset featured more anti-GAD65 (particularly in subjects with two alleles of HLA-DR3/DQ2 haplotypes, although the HLA genetic background seems more heterogeneous in this subgroup).^14,15^ It seems however that the faster clinical progression in early seroconverters reflects the earlier age of the first AAb appearance rather than an anti-insulin vs. anti-GAD65 pattern.^16^ There are other gene variants, environmental exposures and biomarkers that further characterize patients, and when all these characteristics are considered together, they can delineate discrete subtypes termed endotypes,^13,17^ whereby certain characteristics often tend to correlate with one another in subsets of patients. Two patterns correlating HLA haplotype and AAb type have been noted so far in groups of patients:^13^ one associating HLA-DR4(-DQ8) and anti-insulin as predominant and/or first and early appearing AAb, and another associating HLA-DR3(-DQ2) and anti-GAD65 as the predominant and/or first AAb (though appearing later in childhood).^14,18^ However, it should be acknowledged that these endotypes do not encompass the whole range of heterogeneity of T1D.

The NOD mouse is a rare example of a polygenic and spontaneous model of autoimmune disease, and shows many commonalities with human T1D, including a large overlap in genetic susceptibility features, the β-cell antigens targeted by T cells and other autoimmune conditions.^3,19^ However, although it has been used extensively in T1D preclinical studies, it has been criticized for limited translatability of the efficacy of immunotherapies to T1D patients. This shortfall is related to the fact that the NOD mouse, as an inbred and genetically homogeneous strain, simply cannot represent the heterogeneity of the human T1D population. Interestingly, NOD mice have a unique MHC class II haplotype (I-A^g7^) that is highly similar to HLA-DQ8,^20^ and they only develop anti-insulin AAb consistently.^21^ Both I-A^g7^ and DQ8 are known to present the same key epitope of insulin (B9:23), which was found to be essential for T1D to develop in NOD mice.^22^ Therefore, the NOD mouse may constitute a reasonable model for the human endotype featuring HLA-DQ8 and anti-insulin, until humanized mice can be produced to model other endotypes.^23^

In clinical trials that did not achieve their target, *post-hoc* data analyses were performed, whereby one objective was to determine what the best responders had in common. In the case of ASITs, it turns out that the HLA haplotype and/or the AAb profile could be indicators of responsiveness. In the Diabetes Prevention Type 1 (DPT-1) trial testing oral insulin to induce tolerance, protection was not significant when comparing all patients in treatment and placebo groups, but became significant when only patients with anti-insulin AAbs above 80 nU/mL were considered.^24^ This suggests that patients who have a more pronounced AAb reactivity to an antigen may be more responsive to this antigen in ASIT. Mere positivity for the AAbs may not be correlated with responsiveness to therapy, as confirmed by a follow-up trial.^25^ Across three trials involving the delivery of alum-formulated GAD65, it was generally observed that patients with the HLA-DR3-DQ2 haplotype were responsive, particularly if they also did not have the HLA-DR4-DQ8 haplotype.^26^ These two examples support the idea that patient characteristics, which can be ascertained prior to treatment, can constitute useful predictors of responsiveness. As a result, newer ASIT trials are increasingly using these parameters as inclusion criteria (Table 1), and if this practice improves responsiveness, additional *post-hoc* analyses may identify other parameters that correlate with efficacy.

**Table 1.** Examples of clinical trials for antigen-specific immunotherapy of T1D (current and completed within the past five years).

Intervention	Components of ASITs	Endotype-related inclusion criteria	Status	Trial size	Sponsor	NCT number	Stage
IMCY-T1D-001	Small synthetic peptide (Imotopes^TM^)	HLADR3-positive and/or HLADR4-positive	Completed in 2019	41	Imcyse SA	NCT03272269	Phase 1
IMCY-T1D-002	Small synthetic peptide (Imotopes^TM^)	HLADR3-positive and/or HLADR4-positive	Completed in 2019	30	Imcyse SA	NCT04190693	Phase 1
IMCY-T1D-003	Small synthetic peptide (Imotopes^TM^)	HLADR3-positive and/or HLADR4-positive	Recruiting	108	Imcyse SA	NCT04524949	Phase 2
Tolerogenic Dendritic Cell Vaccine PIpepTolDCs	Proinsulin Peptide (C19-A3)	Positive for DRB1 × 04:01, *04:02 and/or *04:04 allele but without the protective HLA-DRB1 × 15:01-DQA1 × 01:02-DQB1 × 06:02 haplotype	Recruiting	7	City of Hope Medical Center	NCT04590872	Phase 1
C19-A3 GNP	C19-A3 proinsulin peptide coupled to gold	Positive for DRB1 × 0401 allele	Unknown	8	Cardiff University	NCT02837094	Phase 1
GAD-Alum	rhGAD65-alum (Diamyd®), Etanercept and Vitamin D	Positive for GAD65 antibodies	Completed in 2019	20	Johnny Ludvigsson (Linköping University)	NCT02464033	Phase 2
GABA-GAD	GAD and GABA	Positive for GAD65 antibodies	Completed In 2022	101	University of Alabama at Birmingham	NCT02002130	Phase 1
GAD-alum (Diamyd®)	rhGAD65 adsorbed to Alhydrogel	HLA DR3-DQ2 haplotype	Recruiting	6	Linköping University	NCT05351879	Phase 1 Phase 2
GAD-alum (Diamyd®)	rhGAD65, formulated in aluminum hydrogel	High GAD antibody titers (>190 U/ml)	Completed in 2022	14	Norwegian University of Science and Technology	NCT04262479	Phase 2
GAD-Alum (Diamyd®)	GAD-Alum (Diamyd) combined with Vitamin D and Ibuprofen	Elevated GAD65 antibodies	Completed in 2017	60	Johnny Ludvigsson (Linköping University)	NCT01785108	Phase 2
GAD-Alum (DIAGNODE-1)	Intra-lymph node rhGAD65 Alum + Vitamin D	Positive for GAD Antibodies	Completed in 2019	12	Johnny Ludvigsson (Linköping University)	NCT02352974	Phase 1
GAD-Alum DIAGNODE-2	Intra-lymph node Alhydrogel®-formulated rhGAD65	Positive for GAD65 Antibodies	Completed in 2021	109	Diamyd Medical AB	NCT03345004	Phase 2
DIAGNODE-3	Intra-lymph node rhGAD65 formulated in Alhydrogel®	HLA DR3-DQ2 haplotype and positive for GAD65 antibodies	Recruiting	330	Diamyd Medical AB	NCT05018585	Phase 3
DIAPREV-IT2	Alum-GAD (Diamyd®) and Vitamin D3	Positive for GAD65 Antibodies plus at least one other	Terminated In 2019	26	Lund University	NCT02387164	Phase 2
MultiPepT1De	Multiple Islet Peptides	HLA-DR4 (DRB1 × 0401) genotype	Completed in 2018	27	King’s College London	NCT02620332	Phase 1
TOL-3021	Plasmid for the hINS gene	At least one of GAD65, IA-2, ZnT8, or insulin antibodies	Active, not recruiting	78	Tolerion, Inc.	NCT03895437	Phase 2
TOPPLE T1D	PPI, TGF-β1, IL-10 and IL-2	At least one diabetes-related autoantibody present	Recruiting	48	National Institute of Diabetes and Digestive and Kidney Diseases (NIDDK)	NCT04279613	Phase 1

rhGAD65 (recombinant human glutamic acid decarboxylase), GAD (glutamic acid decarboxylase), GABA (gamma-amino butyric acid), alum (aluminum hydroxide), HLA (human leukocyte antigens), PPI (preproinsulin), TGF-β1 (transforming growth factor beta 1) and IL (Interleukin).

In sum, the success of ASIT for the treatment of T1D and other autoimmune diseases will benefit from being employed in a targeted manner, not only at the cellular level (targeting only autoreactive lymphocytes), but also at the individual level (tailored to endotype groups). Furthermore, we propose that the NOD mouse should be regarded and utilized more as a disease endotype model, and that promising treatments be next evaluated in the HLA-DR4-DQ8 endotype group, which is prominent in the T1D patient population.

## Why use peptides over full antigens?

As the studies described above used full proteins, the nature and number of epitopes that are ultimately presented on each HLA haplotype is not known. Because T cells selected as natural Tregs tend to have distinct T cell receptors (TCRs) from conventional T cells,^27^ some MHC/peptide complexes may be more likely recognized by Tregs, in which case epitopes can be considered as “useful” for eliciting regulation. Peptides that engage conventional T cells may also be useful if they elicit deletion, anergy or conversion to peripheral Tregs, but that would depend more on the context of presentation rather than the nature of the peptide. Considering the full array of peptides that can be presented on different HLA haplotypes from a protein antigen, it is tempting to speculate that more useful GAD65 peptides are presented on HLA-DR3-DQ2 than on HLA-DR4-DQ8, leading to a better outcome in the GAD65-alum studies. Moreover, some peptides may not be as useful if they interfere with the display of useful peptides by competing for HLA occupancy. Thus, there may be an advantage of using a selection of peptides over the full antigen if these peptides are well-characterized and anticipated to preferentially stimulate Tregs and/or engage low-affinity T cells more effectively under tolerogenic conditions. Because of the ability of Tregs to suppress T cells across multiple specificities within and across antigens, provided that those other epitopes are displayed by the same antigen-presenting cells (APCs), it is not necessary to attempt to cover all possible epitopes in ASITs (Figure 2).

The array of epitopes that have been identified from T1D-relevant proteins is constantly growing, although their level of characterization and validation is highly varied. A search in the Immune Epitope Database (IEDB) yielded 8,234 MHC class I and 3,146 MHC class II human linear epitopes, the great majority of which were eluted from MHC. A more focused search on epitopes discovered by T cell assays returned 182 MHC class I and 401 MHC class II epitopes, as of the time of writing (Table 2). These were typically identified by stimulating islet-infiltrating T cell clones with peptide libraries encompassing major β-cell antigens.^28–30^ The recent and comprehensive review by James et al. addresses most of these epitopes, providing a detailed analysis of their antigen source and HLA-restriction.^31^ About 75% of epitopes recognized by CD8+ T cells are derived from insulin, ZnT8 and GAD65, and about the same percentage is restricted to the common HLA-A2 allele. About 80% of epitopes recognized by CD4+ T cells are derived from GAD65, insulin and IA-2, 1/3 are restricted to HLA-DR4 and 1/4 are restricted to HLA-DQ8. Still, these numbers do not reflect the reality but rather the current knowledge, which is itself biased toward those haplotypes most commonly found in T1D patients. The IEDB also provides detailed information about TCRs recognizing some of these epitopes on specific HLA haplotypes (V region usage and CDR3 sequence) sufficient to produce TCR-transgenic T cell transductants that may be used to validate the presentation of epitopes from various ASIT delivery platforms in vitro or using in vivo models.^23^

**Table 2.** Human T cell linear epitopes deposited in the Immune Epitope Database (IEDB) and validated by T cell assays, and corresponding TCRs identified. Filters applied: linear structure for epitope structure; Homo sapiens for both organism and host; type 1 diabetes mellitus for disease; include positive T cell assay; exclude B cell assays and MHC assays; further filtered on MHC class I, class II or specific HLA protein complex as indicated in the table). A more limited number of epitopes (third column) were from assays in which the TCR sequence of responding T cells was characterized (at least one chain in fourth column; or both alpha and beta chains in fifth column). One epitope can be recognized by multiple T cell clones (TCR alpha/beta pairs). Searches including both T cell and MHC assays (eluted peptides) yielded 8,234 epitopes for HLA class I and 3,146 epitopes for HLA class II. Data are accurate as of 11/02/2022.

MHC	Total epitopes	Epitopes recognized by TCRs	Number of TCRs identified	Number of paired TCRs (unique clones)	Main antigens
**HLA class I**	**182**	**24**	**135**	**79**	
HLA-A	125	17	122	66	
HLA-A *02:01	*110*	*14*	*120*	*64*	*ZnT8 (31), insulin (26), GAD65 (9), IA-2 (8), IGRP (6)*
HLA-A *03:01	*11*	*0*	*0*	*0*	
HLA-B	13	6	6	6	Insulin (6), GAD65 (6)
HLA-C	4	3	7	7	Insulin (4)
**HLA class II**	**401**	**34**	**182**	**47**	
HLA-DP	3	1	1	1	IA-2 (2), insulin (1)
HLA-DQ	66	24	35	35	
*HLA-DQB1 × 02:01*	*6*	*6*	*6*	*6*	*Insulin (6)*
*HLA-DQB1 × 03:02*	*53*	*20*	*29*	*29*	*Insulin (33), IA-2 (8), GAD65 (4), IAPP (2), HIPs (2)*
HLA-DR	119	12	148	13	
*HLA-DRB1 × 01:01*	*5*	*1*	*1*	*1*	*GAD65 (4), insulin (1)*
*HLA-DRB1 × 03:01*	*3*	*0*	*0*	*0*	*IGRP (2), insulin (1)*
*HLA-DRB1 × 04:01*	*75*	*4*	*140*	*6*	*GAD65 (29), insulin (11), IA-2 (11), HIPs (6), hexokinase-4 (5), IGRP (4)*
*HLA-DRB1 × 04:02-05*	*7*	*2*	*2*	*2*	*Insulin (4), GAD65 (3)*

The identification of neoepitopes targeted by diabetogenic T cells has been a major advance in the T1D field. Aside from “native” peptides resulting from the simple proteolytic cleavage of known antigenic proteins, there are other types of peptides that have been hypothesized to play a significant role in expanding the pool of pathogenic T cells and/or in targeting them in ASIT:^32,33^

1. Peptides resulting from the post-translational modification (PTM) of native peptides (deamidation, citrullination, etc.).^32^
2. Peptides resulting from the fusion of peptides from different antigens (hybrid peptides) creating new epitopes centered on the junctions of the original two peptides.^34–36^
3. Peptides encompassing new junctions produced from alternatively spliced mRNA occurring in islets but not in the thymus and other lymphoid tissues.^37–40^
4. Peptides originating from defective ribosomal products (DRiP) whose expression may be increased under the influence of T1D-associated gene polymorphisms.^41^
5. Artificial peptides (often termed mimotopes or altered peptide ligands) that either correspond to native peptides mutated to help fix anchoring on MHC in a particular conformation or register to enhance recognition by a specific type of T cells^42^ or that were identified for their ability to stimulate islet-infiltrating T cells of unknown specificity.^43^

These neoepitopes greatly increase the antigenic display that make up the specificities of diabetogenic T cells to the point that the use of a single protein may appear preposterous, no matter how many MHC-binding epitopes that single protein may produce. It is not yet clear whether certain types of neoepitopes are associated with specific endotypes, and whether some are early drivers of the disease or appear during later stages of disease. Because some of these neoepitopes may arise under certain stress or inflammatory conditions, it is probable that they are not produced and presented under steady state conditions and that tolerance to these neoepitopes is limited. Moreover, they are not templated in the genome and may therefore be preferentially recognized as “non-self.” Thus, the inclusion of neoepitopes in ASIT to build this tolerance may prove important. Using mimotopes, as well as native epitopes that are generally ignored,^44^ increases the chance of targeting antigen-specific T cells that are more naïve (and likely easier to manipulate phenotypically) and possibly more frequent.

There are other advantages of using peptides over proteins for ASIT:

1. It is easier to synthesize peptides than to produce recombinant proteins. Peptides can be synthesized with specific mutations or chemical modifications that correspond to PTMs, and the chemical synthesis process circumvents issues of biological contaminants from protein-producing cells.
2. The use of peptides is more amenable to customizing the treatment of T1D patients grouped by endotypes, using a “mix & match” approach.
3. Some delivery strategies are more compatible with the use of peptides than proteins.
4. Despite their shorter in-vivo half-life compared to proteins, potency and persistence of peptides can be improved by chemical modifications.^45^

Moreover, just like proteins can be encoded by DNA vectors (as in DNA vaccines for T1D^46–50^), it is possible to express a wide variety of epitopes from a single vector,^51–53^ with the same previously mentioned advantages. Endogenously encoded proteins and polypeptides have the added benefit of allowing the host cells to perform PTMs on them naturally under the appropriate conditions. DNA vectors are also cheaper to produce, and while the efficiency of DNA delivery remains relatively limited, one molecule generates many mRNA molecules, which in turn produces hundreds of proteins or polypeptides.^54^

One limitation of using peptides over proteins is that they are unable to engage autoreactive B cells that only recognize conformational epitopes. It is unclear to what extent targeting β-cell antigen-specific B cells for tolerance can help in blunting the overall autoimmune response, but one may speculate that anergized B cells serving as APCs may contribute to tolerization of T cells and/or regulatory B cells may help suppress autoreactive T cells. Several studies have suggested a beneficial involvement of regulatory B cells in treatment of T1D.^55–58^

## What immune biomarkers and epitope characteristics may be used for customization?

The concept of endotype being relatively new, associations between patient characteristics and targetable antigen specificities are yet to be made. AAbs are reliable biomarkers that are routinely measured. While the number of AAbs informs on the relative risk of progressing to overt disease, the relative titer of each AAb may provide clues on dominant immune reactivities. As AAb-producing B cells require initial help from antigen-specific CD4+ T cells, this can provide an indirect indication of dominant T cell responses. However, it remains to be seen in other trials if the DPT-1 trial *post-hoc* results are generalizable and if AAb titers can be used for antigen selection to achieve significant responsiveness.

A more direct measurement of T cell specificities would be to perform multiplex MHC multimer analysis to determine whether certain antigen-specific T cell populations are expanded in the circulation, possibly delineating populations that are worth targeting. In this approach, MHC multimers are used, sometimes in pairs (with two different detectable conjugates) for most accurate detection of antigen-specific T cells, using conventional or spectral flow cytometry (fluorochrome-labeled), mass cytometry (heavy metal-labeled) or DNA sequencing (DNA barcode-labeled).^59^ In T1D, approaches with combinatorial MHC multimers validated the existence of autoreactive CD8+ T cells specific to a wider array of antigens than previously anticipated.^40,60^ The same approach can be used to analyze CD4+ T cells,^61^ but has yet to be applied for T1D-relevant specificities. There are, however, several challenges to this approach:

1. Detection of autoreactive T cells is complicated by the fact that many of their TCRs are of relatively low affinity and thus signal may be weak.
2. This is an expensive approach. Even with high-throughput methods utilizing DNA barcodes, the high cost of MHC multimer manufacturing dramatically limits how comprehensive the analysis panel can be. However, the panels would be limited to using only MHC multimers matching the HLA haplotype of the tested individuals.
3. The frequency of relevant antigen-specific T cells is very low in the blood, and it remains debated whether circulating autoreactive T cells provide a useful reflection of those infiltrating the target tissue (pancreatic islets in the case of T1D).

Studies using MHC multimers also reported that autoantigen-specific T cells are found in the circulation of healthy individuals, typically with similar frequencies,^40,60,62,63^ which suggests that negative selection in the thymus may be limited to the highest affinity autoreactive T cells in all individuals, and that in T1D patients, it is mainly a defective regulation of the remaining T cells that allows them to be activated and become pathogenic.^64^ Beyond detecting those T cells, it may be more important to determine their phenotype (i.e., whether these circulating T cells are more activated and antigen-experienced). Again, in MHC multimer-based analyses of circulating autoreactive CD8+ T cells comparing control and T1D donors, differences are difficult to pinpoint. Recently, a method combining DNA-barcode-labeled MHC tetramers, CITE-seq and TCR-seq (termed TetTCR-SeqHD) was employed to profile CD8+ T cells in control and T1D individuals.^65^ While this approach provides a much deeper profiling of these rare T cells at the clonal level (thanks to TCR sequences), it is challenging to apply on a large scale (broad antigen-coverage by MHC multimers and large number of patients for screening purposes).

Another assessment of T cell specificities can be done by restimulating PBMCs with specific peptides and measuring cytokines produced in response using ELISPOT assays. In such study using insulin mimotopes, CD4+ T cells from both control and T1D donors generated IFN-γ+ spots.^66^ However, the CD4+ T cells from control donors produced IL-10+ spots more frequently than those from T1D donors,^66^ indicating that differences in phenotypes may be discernable to ascertain the relevance of certain antigen specificities.

Regardless of the approach used, building a comprehensive immune profile for patients and attempting to identify major/driver antigens may be a daunting task at this time, and greater automation and cost reduction will be required. While having more options (epitopes/antigens) to choose from may be helpful, using a large number of peptides for immunotherapy may not be required. The question is then: how many epitopes are enough in ASIT to achieve control of the disease? Several studies in NOD mice suggest that one or few peptides may be enough to achieve significant protection from disease onset, likely through bystander suppression and infectious tolerance (Figure 2), and these peptides were generally strong binders (Table 3). In humans, clinical trials have been conducted with up to six HLA-DR4-restricted insulin- and IA2-derived peptides, which were believed to engage Tregs (Table 1). These studies have in common that all peptides used targeted CD4+ T cells, with the rationale that induction/stimulation of antigen-specific Tregs can suppress across specificities, and with the presumption that the number of functional Tregs may be more important than their diversity.

**Table 3.** Antigen-specific approaches pursued by biotech companies and academic laboratories.

Form of delivery	Approach	Disease	Peptide used	Route	Company/University	Stage	Link or Reference
Free peptides	Mimotope with stronger MHCII-binding	T1D	Insulin_9-23_ mimotope	SC	Helmholtz Zentrum München	Pre-clinical	Refs.^67,68^
	Apitopes®: tolerogenic peptides	MS	Four myelin-derived peptides	ID	Worg Pharmaceuticals	Phase 2	http://www.worgpharma.com
	Imotopes®: thioredoxin-Ag to augment pMHC-TCR interactions	T1D, MS	PPI-derived peptide, MOG-derived peptide	SC	Imcyse/University of Leuven	Phase 2	https://www.imcyse.com, Ref.^69^
	Multiple insulin peptides	T1D	PPI_C13-C32_, PPI_C19-A3_, PPI_C22-A5_, IA2_718-36_, IA2_752-75_, IA2_855-67_	ID	King’s College London	Phase 3	Ref.^70^
Conjugated peptides	Galactose or N-acetyl-galactosamine-terminating Ags	T1D, MS	p31 mimotope, myelin-derived peptides	IV	Anokion	IND, Phase 1	https://anokion.com
	Soluble Ag Arrays (SAgAs)	T1D	p79 mimotope, 2.5HIP	SC	University of Kansas/Columbia University	Pre-clinical	Ref.^71^
	Abs conjugated peptides targeting DCs (aDEC, aDCIR)	T1D	p31 mimotope	IP	NIH	Pre-clinical	Ref.^72^
Peptide carrying-NPs	PLGA co-delivery of Ag and rapamycin	PBC	PDH-E2-derived peptide	IV	Selecta Biosciences	Pre-clinical	https://selectabio.com
	Liposome or gold NPs co-delivery of Ag and AhR ligand	T1D, MS	PPI-derived peptides, MOG_35-55_	SC, IV	Antolrx/Pfizer	Pre-clinical	https://antolrx.com, Ref.^73^
	Synthetic HDL to deliver Ag	T1D	NOD Ag	SC	Evoq Therapeutics	Pre-clinical	https://www.evoqtherapeutics.com
	pSER liposomes taken up as apoptotic cells	T1D	Ins_90-110_ and Ins_25-54_	IP	Ahead Therapeutics/Universitat Autonoma de Barcelona	Pre-clinical	https://www.aheadtherapeutics.com, Ref.^74^
	PLG NPs uptake by phagocytes	T1D	2.5HIP	IV	University of Colorado/Northwestern University/Cour Pharmaceuticals	Pre-clinical	https://courpharma.com, Ref.^75^
	Super paramagnetic iron oxide NPs (Spions®)	PV	Desmoglein 3-derived peptides	IV	Topas Therapeutics	Phase 1	https://topas-therapeutics.com
	NPs made of polymerized ursodeoxycholic acid	T1D	INS (protein, adaptable to peptides)	Oral	Toralgen/Yale University	Pre-clinical	https://toralgen.com, Ref.^76^
mRNA carrying-NPs	Liposome with mRNA-encoding Ags	MS	MOG_35-55_	IV	BioNTech/University of Mainz	Pre-clinical	Ref.^77^
DNA vectors	Plasmid encoding Ag and targeting lysosomal compartment	T1D	p31 mimotope	IV	University of Barcelona	Pre-clinical	Ref.^78^
	Plasmid encoding Ag, TGF-β1, IL-10, IL-2	T1D	PI (protein, adaptable to peptides)	SC	Novo Nordisk	Phase 1	Ref.^49^
	Plasmid encoding multiple Ags (Endotope)	T1D	Ins_B9-23_ p8E and p8 G mimotope, p79 mimotope, Ins_B9–23,_ GAD65_286-300_, GAD65_524-543_, Ins_B15–23,_ IGRP_206-214_	IM, ID	Columbia University	Pre-clinical	Ref.^53^
Modified bacteria	Lactococcus lactis expressing IL-10, Ag	T1D	PI (protein, adaptable to peptides), GAD_375-575_	Oral	Precigen Actobio/University of Leuven	Phase 1/2	https://precigen.com, Refs.^79,80^
	Bacterium-like particles	T1D	IA-2ic (protein, adaptable to peptides)	Oral	Wuhan University	Pre-clinical	Ref.^81^
Modified erythrocytes	RBCs (or PBMCs) coupled with Ag	MS	Seven myelin-derived peptides	IV	Cellerys/Novartis/University of Zurich	Phase 2	https://www.cellerys.com
	RBCs loaded with Ag	T1D	p31 mimotope	IV	SQZ Biotech	Pre-clinical	https://sqzbiotech.com
	RBCs transduced with Ag	T1D	p31 mimotope	IV	Rubius Therapeutics	Pre-clinical	https://www.rubiustx.com
Soluble pMHC complexes	Dimeric MHC II-peptide chimera	T1D	GAD65_217-230_	IV	Mount Sinai School of Medicine	Pre-clinical	Ref.^82^
APCs/APNPs	Tolerogenic DCs	T1D	PI_C19-A3_	ID	Leiden University, City of Hope Medical Center	Phase 2	Ref.^83^
	Iron oxide NPs with large density arrays of pMHC (Navacims®)	T1D, MS, PBC	GAD65_555-567_, PI_76–90,_ IGRP_13-25_, MOG_38-49_, PDH-E2_166-181_	IV, SC	Parvus Therapeutics/University of Calgary	Pre-clinical	https://parvustx.com, Ref.^84^

Ag, Antigen; NP, nanoparticle; APCs, antigen-presenting cells; APNP, antigen-presenting nanoparticles; aDEC, anti-DEC205; aDCIR, anti-DCIR2; PLGA, poly(lactic-co-glycolic acid); pSER, phosphatidyl-serine; RBCs, red blood cells; DCs, dendritic cells; T1D, Type 1 diabetes; MS, multiple sclerosis; PBC, primary biliary cirrhosis; PV, pemphigus vulgaris; MOG, myelin oligodendrocyte glycoprotein; PI, proinsulin; PPI, preproinsulin; NOD, non-obese diabetic; IA, insulinoma-associated; HIP, hybrid insulin peptide; INS, insulin; PDH-E2, pyruvate dehydrogenase-E2; GAD, glutamic acid decarboxylase; IGRP, islet-specific glucose-6-phosphatase catalytic subunit-related protein; SC, subcutaneous; ID, intradermal, IV, intravenous; IP, intraperitoneal.

The choice of peptides may also be based on their properties, beyond their ability to bind specific HLA haplotypes. As alluded to earlier, certain PTMs and mutations can affect the position and strength of binding, but they can also enable recognition by additional and otherwise unresponsive populations of T cells. The outcome of the T cell response can be influenced by the affinity of TCR binding to the peptide/MHC complex, and similarly, by the avidity of TCR recognition, which can be modulated by varying the dose of peptide administered. For example, when two epitopes stimulating the same T cell clone (BDC2.5) were tested in soluble antigen array (SAgA) form in NOD mice, the stronger one (p79 mimotope^43^ primarily induced a Tr1 (IL-10-producing) type of Tregs, with a lower dose needed to stimulate Foxp3+ Tregs, whereas the 2.5HIP peptide^34^ (about 10-fold weaker than p79 in vitro) at the same dose only stimulated Foxp3+ Tregs.^71^ The two peptides did not change the frequency of IFN-γ+ antigen-specific T cells, but rather induced IL-10+ T cells or stimulated Foxp3+ Tregs. In some cases, chemical modifications may change the properties of the peptide to functionally alter the phenotype of responding T cells. For example, Imcyse’s Imotopes^TM^ are HLA class II-binding peptides fused with a small peptide motif with oxido-reductase activity, which confers cytolytic activity to the activated CD4+ T cells, resulting in the killing of autoantigen-presenting cells and autoreactive T cells around them^69^ (Table 3).

Once pre-filtering based on known HLA restriction is accomplished, epitopes may be selected based on the source antigen (with a focus on one or few antigens) and on how well they are characterized. Epitopes that are simply predicted or eluted from MHC do not provide as much confidence as those that have been demonstrated to induce a T cell response. In particular, those found to be recognized by islet-infiltrating T cells would be prioritized as highly relevant, and the list is growing,^85^ especially with recent efforts on identifying and validating neoepitopes using T cells from pancreatic islets and lymphoid tissues rather than blood. Once a group of disease-relevant epitopes has been defined for a specific HLA haplotype, could a mix of epitopes covering multiple antigens be used as a blanket treatment? In other words, if a patient’s autoimmune response is dominated by reactivity against insulin, would introducing other antigens by ASIT be beneficial or detrimental? This is a question that has not yet been addressed because patients have so far only been treated with one antigen at a time, except in the MultiPepT1De trial in which they received peptides from both proinsulin and IA-2 (Table 3),^70^ and there is only one spontaneous mouse model, representing one endotype, available for testing. If epitopes from other antigens induce Tregs and all epitopes tend to be naturally presented by the same APCs, then one may speculate that covering many antigens would have a beneficial outcome. In contrast, if epitopes are presented under inappropriate conditions (leading to preferential stimulation of effector T cells), then adding other epitopes could be detrimental. Blanket coverage with diverse peptides from multiple antigens is costly, unless those peptides can all be produced from a single molecule (DNA or mRNA)^51^ or recombinant protein.^86^ For example, the Endotope platform enables expression of multiple MHC class I and II restricted epitopes from a DNA vaccine, whereby MHC class II epitopes are separately funneled from cytosol to endosome.^51,52^ An Endotope DNA vaccine including native epitopes and mimotopes and covering at least three different antigens was comparable in efficacy to a proinsulin-encoding DNA vaccine in preventing disease in NOD mice (insulin-dominated endotype).^53^

Another outstanding question that remains unanswered is whether it is sufficient to target CD4+ T cells (if resulting in suppressive antigen-specific Tregs) in a disease like T1D wherein CD4+ and CD8+ T cells play a critical role. While there is evidence that some ASITs can result in deletion of autoreactive CD8+ T cells in patients, this appears to be limited to those reactive to the administered antigen.^47^ The current favored approach for epitope-based ASIT is to achieve a sufficient level of suppression with a minimal set of key HLA class II epitopes that would be effective across all patients within the same HLA haplotype group. However, given that Treg stability may be an issue in some T1D patients, targeting and eliminating certain CD8+ T cells that are consistently expanded in T1D patients may function as a debulking therapy and increase protection from disease. The use of disease-irrelevant epitopes that activate Tregs and are co-delivered with disease-relevant epitopes may also be considered. For example, Tregitopes are IgG epitopes recognized by Tregs, which become mobilized around diabetogenic T cells when Tregitopes are co-delivered and co-presented with preproinsulin peptides, resulting in disease improvement.^87^

## How can we deliver peptides to suitable locations and APCs in vivo?

Once epitopes are selected, the next challenge is to find suitable modalities to deliver them to appropriate locations where APCs and their environment are conducive to tolerance. Table 3 summarizes delivery strategies by companies and academic laboratories. The simplest form of delivery is by injection of peptides in soluble form. These peptides are most vulnerable to extracellular degradation and may diffuse widely (unless injected in a dense tissue), potentially resulting in relatively low density of epitope presentation. Multimerization of peptides or formulation in small nanoparticles (<200 nm) achieves higher molecular weights that are more conducive of draining through lymphatics to achieve more specific accumulation in T cell-rich lymph nodes, and possibly spleen. Some nanoparticles, based on their chemical composition and net charge, are preferentially taken up by specific APCs that are tolerogenic in nature (e.g., marginal zone macrophages, liver sinusoidal endothelial cells).^88,89^ Larger microparticles (as well as large macromolecules such as DNA plasmids) remain trapped in the tissue in which they are injected and rely on tissue APCs with the capacity to migrate to draining lymph nodes.^52,90^ Small and large particles have also been used to remodel the environment of the antigen uptake and/or presentation sites (with nanoparticles influencing lymph node environment^91^ and microparticles modulating the inoculation site,^92^ for example). Table 3 provides examples illustrating the variety of nanoparticle formulations that differ in size, properties, and content (co-delivery of drugs along with antigens). While some formulations are compatible with oral delivery,^93^ antigens have also been delivered inside commensal bacteria for oral tolerance.^79,80,94^ Alternative to using nanoparticles, peptides may be grafted onto natural polymers (hyaluronic acid, to produce SAgA),^71^ conjugated to glycopeptides that enhance uptake in liver^95^ or to antibodies against APC surface markers^72–96–98^ or associated with erythrocytes that are taken up by tolerogenic splenic macrophages by efferocytosis.^99^ Peptides may be expressed from DNA or mRNA vaccines, which often require formulation into nanoparticles or liposomes to improve the delivery and control the biodistribution.^77,100^ Peptides can also be provided as already presented on MHC molecules, either on the surface of tolerogenic dendritic cells^83^ or on the surface of small nanoparticles,^84^ the latter providing TCR stimulation without any costimulation to tolerize T cells. These can be used for direct presentation to T cells following administration, without requiring any expression and/or processing. Finally, instead of inducing tolerance or eliciting Tregs with specific epitopes, it is possible to use epitope-specific TCRs to engineer Tregs for cell therapy.^101^ This approach requires identification of islet antigen-specific TCRs from islet-infiltrating T cells with the appropriate HLA restriction and any Treg, and the patient’s T cells can be engineered ex vivo to replace the preexisting TCR with a new disease-relevant TCR, then expanded prior to re-infusion. The use of chimeric antigen receptor (CAR) Tregs affords the additional advantages of whole antigen recognition rather than a specific peptide/MHC complex and independence from HLA haplotype,^101^ although this may also be a limitation when the antigen is not a surface antigen (like many β-cell antigens) and different targets are needed. The issue of Treg stability is crucial as conversion of transferred epitope-specific Tregs to effector T cells in vivo could exacerbate the disease, and additional engineering methods or treatment to stabilize Treg function are actively sought.

## Thoughts on ensuring appropriate conditions of epitope presentation

When antigenic proteins or peptides are administered to individuals, it is difficult to tightly control the type of APCs that participate in their presentation, and the tissue environment in which these antigens are taken up which influences the phenotype of these APCs. Sites like liver and gut-associated lymphoid tissues generally provide a more tolerogenic environment,^102,103^ whereas in spleen and lymph nodes, tolerance induction is more dependent on the type of APCs. When professional APCs (cells capable of maturing to elicit immunogenic responses) are involved, it is desirable to target non-inflamed environments and ensure that nothing administered with the antigens (e.g., chemical modifications, nanodelivery formulations, DNA/RNA motifs when expressing antigens) acts as immunogenic adjuvant while maximizing exposure to the extensive pool of circulating T cells. Specific dendritic cell populations (e.g., DEC205+ vs DCIR2+) have been targeted with specific antibodies that were conjugated with peptides^72^^–96–98^ and reported to promote tolerance. Alternatively, nonprofessional APCs have been targeted, including hepatocytes,^95,104^ liver sinusoidal endothelial cells (e.g., Topas’ SPIONS^TM^, Table 3), and lymph node stromal cells.^105^ For example, hepatocyte-restricted delivery can be achieved using N-acetylgalactosamine-modified antigens^95^ (approach used by Anokion, Table 3) or using liver-specific promoter coupled with micro-RNA targets restricting expression in non-hematopoietic cells in the case of transgene-encoded antigens.^104^ When the type of APCs cannot be restricted, codelivery of immunomodulatory drugs (e.g., rapamycin^76,106^ or cytokines (e.g., IL-10)^79,107^ with antigens helps maintain the tolerogenic function of APCs. Importantly, a single DNA vaccine encoding antigen and multiple cytokines (IL-2, IL-10 and TGF-β) has been validated^49^ and is being tested clinically, providing a 4-in-1 drug option that is easy and economical to manufacture.

A more broadly distributed exposure of epitopes via systemic delivery may help with engaging more autoreactive T cells in a shorter time frame, while local delivery that results in presentation at limited sites may require more prolonged exposure or more frequent administrations. Persistence of self-antigen presentation appears critical for the induction of immune tolerance (anergy/exhaustion and deletion) in CD4+ T cells^108–115^ and CD8+ T cells,^116,117^ regardless of whether APCs are DCs^112,114^ or B cells.^110,111^ In fact, prolonged antigen exposure can lead to tolerance even when APCs were initially in an immunogenic state.^111,117^ However, in the case of DCs, T cells can maintain effector functions after at least 3.5 days of continuous antigen presentation,^112^ suggesting that antigen exposure should be sustained longer. These studies used continuous or regulated transgenic expression of antigens, but in ASIT, the frequency of delivery and duration of antigen presentation after each delivery differ between delivery modalities, resulting in continuous or intermittent antigen exposure. Peptides have a short half-life, whereas those delivered via nanoparticles persist longer. Multivalent delivery of peptides as SAgAs also significantly prolonged antigen presentation in vivo.^71^ Controlled release of peptides can be achieved by osmotic pumps,^67^ but peptides remain subject to extracellular degradation, resulting in sustained yet limited peptide delivery. DNA vaccines can sustain antigen presentation for at least two weeks in NOD mice,^53^ but exposure is relatively limited (local) and administration once or twice a week was required to achieve sufficient T cell engagement and significantly block disease progression.^49,53^ While frequent administrations may be required at first to achieve a sufficient level of tolerance, treatments may potentially be discontinued or reduced in dose or frequency for maintenance. The route and frequency of administration represent challenges for implementation and compliance in patients, for example if frequent injections are needed. When frequent administrations are required to maintain a sufficient and sustained exposure of epitopes to autoreactive T cells, the oral route stands out as the most attractive for patients if adequate antigen protection and T cell responses can be obtained.

## Outlook: what are major challenges to overcome?

The field of ASIT in T1D (and other autoimmune diseases) has witnessed significant advances with new methods that accelerate the discovery and validation of disease-relevant epitopes, new biomarkers and signatures. These strides enable the stratification of patients into endotypes and the prediction of responsiveness to therapy. In parallel, a burst of biotech activities that follow a diverse and promising array of approaches (Table 3) to achieve adequate delivery and presentation of epitopes for tolerance has taken place.

There remain a series of challenges that most approaches are confronted with and will need to be further tackled in the next few years:

1. Biomarkers: How do we validate whether a set of common autoantigens or epitopes is relevant for a specific patient? We need additional and reliable markers that can discern autoreactive T cells in the blood (also detected in healthy individuals) that play an active part in the disease.
2. Models: Preclinical evaluation of ASIT is hampered by the poor representation of patient heterogeneity in animal models. While the NOD mouse model was serendipitously discovered, the current knowledge of T1D patient genetics may inform the engineering of new mouse strains that spontaneously develop T1D. Alternatively, mice hosting human immune systems from T1D patients may be used to recapitulate certain disease characteristics and assess T cell responsiveness to therapy in comparison to control immune systems.^23,118^
3. Combination therapy: Protection through the use of ASIT alone in patients has proved difficult to achieve and tolerance takes time to build up. Inductive treatments that dramatically, temporarily, and selectively disable effector T cell populations (e.g., with targeted biologics) may help reset the immune system before patients are subsequently “retrained” with ASIT to elicit regulation over a more controllable effector population.
4. Treg instability: This remains a major issue in the T1D field. Long-term maintenance of Treg numbers and function is as important as their induction. We need to better understand the signals that control Treg stability and lifespan. Continuous (and tolerogenic) antigen exposure and low-dose IL-2 may be some of the requirements, and ex vivo engineered epitope-specific Tregs might provide the ultimate weapon to potently regulate diabetogenic T cells.
5. Persistence of antigen presentation: For ASIT platforms in which the duration of antigen presentation is shorter than the frequency of administration, antigen exposure is intermittent. How this affects the tolerization process and T cell responses as compared to continuous exposure remains to be investigated more closely and deciphered to understand the requirements for durable tolerance.
6. Autoreactive T cell heterogeneity: While different flavors of Tregs may be induced, which may work synergistically, effector T cells may be more difficult to curb than previously thought. In addition to possible resistance to regulation by effector T cells,^119,120^ a reservoir of self-replenishing stem-like autoreactive CD8+ T cells was recently uncovered in NOD mice^121,122^ and are likely to exist also in humans,^123^ which may subsist in the background and require its own targeting approach.
